# Endocrine system-related adverse events associated with PD-1/PD-L1 inhibitors: data mining from the FDA adverse event reporting system

**DOI:** 10.3389/fmed.2024.1366691

**Published:** 2024-04-16

**Authors:** Hongxia Shi, Yunhua He, Siyuan Dan, Lin Yang, Jing Wang, Li Chen, Zelian Chen

**Affiliations:** ^1^Department of Pharmacy, West China Hospital, Sichuan University, Chengdu, China; ^2^Department of Pharmacy, Sichuan Mianyang 404 Hospital, Mianyang, China; ^3^Department of Pharmacy/Evidence-Based Pharmacy Center, West China Second University Hospital, Sichuan University, Chengdu, China; ^4^Department of Pharmacology, Faculty of Medicine, University of the Basque Country UPV/EHU, Leioa, Spain

**Keywords:** PD-1/PD-L1 inhibitor, endocrine system, adverse events, FAERS, data mining

## Abstract

**Background:**

Various immune checkpoint inhibitors, such as programmed cell death protein-1 (PD-1) and its ligand (PD-L1), have been approved for use, but they have side effects on the endocrine glands.

**Methods:**

Adverse event reports related to PD-1/PD-L1 inhibitors from the FDA Adverse Event Reporting System (FAERS) from the first quarter of 2019 to the first quarter of 2023 were extracted, and the reported Odds ratio methods (ROR method) and comprehensive standard methods (MHRA methods) were used for data mining and analysis.

**Results:**

A total of 5,322 reports (accounts for 6.68% of the total reports)of AEs in endocrine system were collected, including 1852 of pabolizumab (34.80%), 2,326 of navuliumab (43.71%), 54 of cimipriliumab (1.01%), 800 of atilizumab (15.03%), 222 of duvariumab (4.17%) and 68 of averumab (1.28%). Endocrine system-related AEs were mainly present in men (excluding those treated with pembrolizumab) aged ≥65 years. The ratio of AEs components in the endocrine system for the six drugs was approximately 3–8%. The main endocrine glands involved in AEs were the thyroid (pembrolizumab), pituitary and adrenal (nivolumab), adrenal (cemiplimab, atezolizumab, and avelumab), and thyroid (durvalumab). Most patients experienced AEs between 30 and 365 (mean, 117) days,the median time was 61d. AEs resulted in prolonged hospitalization in >40% and death in >10% of cases after administration of pembrolizumab, nivolumab, or durvalumab.

**Conclusion:**

Men aged ≥65 years should be concerned about endocrine-related AEs. There was a lengthy interval between the use of PD-1/PD-L1 inhibitors and endocrine system-related AEs, but the outcome was serious. Special attention should be given to endocrine system-related AEs when using pembrolizumab, nivolumab, or durvalumab.

## 1 Introduction

The programmed death-1 and programmed cell death-ligand 1 (PD-1/PD-L1) were major immune checkpoint inhibitors. The PD-1/PD-L1 derived drugs were specifically recognizing and blocking immunosuppressive molecules to achieve anti-tumor response, namely enhancing anti-tumor immune response, inhibiting immune evasion, inducing tumor cell death,It’s called immunotherapy for tumors (1–3). The immunotherapy is another important therapy strategy after surgery, chemotherapy and radiotherapy, which has been widely applied in the treatment of melanoma, lung, lymphoma, kidney, endometrial and other tumors (2, 4–6). However, PD-1/PD-L1 inhibitor will enhance over-activated immune cells response to normal cells, resulting in immune-related adverse events (irAEs) in organs or tissues.

Therefore, while benefiting from treatment, cancer patients will also be troubled by irAEs, such as gastrointestinal toxicity, skin toxicity, endocrine toxicity, immune-associated pneumonia, etc. (2), involving multiple systems. Treatment can trigger autoimmune reactions in various ways (e.g., increasing the level of autoantibodies (1)) and then involve multiple glands (e.g., pituitary, thyroid, and adrenal) to cause the corresponding functional disorders. Recent studies have shown that endocrine toxicity is irreversible in 50% of cases (7) and can be life-threatening if not identified and treated appropriately (8–10). The disproportional reporting is most usually employed in adverse drug events signal monitoring, which containing reporting odds ratio (ROR), comprehensive standard (MHRA) and proportional reporting ratio (PRR) methods. Up to date, there are numerous studies have been applied these methods in drug safety investigation (11–13).

Therefore, the proportional imbalance method was adopted in this study, we conducted data mining through the US Food and Drug Administration (FDA) Adverse Event Reporting System (FAERS) database. We focused on AEs reported after the use of PD-1/PD-L1 inhibitors in the endocrine system. We concentrated on the risks and characteristics of AEs caused by these drugs and provided references for further prevention and management.

## 2 Methods

### 2.1 Data sources

FAERS is an open database of anonymous patient health and treatment information that contains information on adverse event and medication error reports submitted to FDA. We used data from the FAERS database. The present study did not involve therapeutic interventions or the collection of human samples and, as such, was exempt from approval from an institutional review board approval. There are seven tables: patient demographic and administrative information, medication information, adverse drug reaction information, information on reporting sources, start and end dates of drug therapy, indications for use/diagnosis, and case deletions.

There are numerous types of PD-1/PD-L1 inhibitors. We included only single-agent preparations and excluded varieties available only on the market in China. As a result, we included six drugs for analyses. Pembrolizumab is a PD-1 inhibitor. Nivolumab is a PD-1 inhibitor. Cemiplimab is a PD-L1 inhibitor of atezolizumab, durvalumab, and avelumab.

### 2.2 Data processing

Cemiplimab was launched in the USA relatively recently (September 2018) compared with the other drugs. Hence, the time range of data extraction in the present study was the first quarter of 2019 to the first quarter of 2023 (17 quarters in total). The search terms (including the generic name and trade name of the drug) we used were “pembrolizumab/Keytruda,” “nivolumab/opdivo/opdualag,” “cemiplimab/libtayo,” “atezolizumab/tecentriq,” “durvalumab/imfinzi,” and “avelumab/bavencio.” The FAERS database is updated each quarter, so published reports will inevitably be duplicated. Hence, reprocessing was done using MySQL 8.0,^1^ as recommended by the FDA. If the CASEID and FDA_DT were identical, then the latest PRIMARYID was selected. If the CASEID and FDA_DT were identical, then the DELETE report was selected from the DELETE table. The FAERS database is encoded using the Medical Dictionary for Regulatory Activities (MedDRA) of the International Council for Organizations of Medical Sciences. Therefore, systematic organ classification (SOC) and the preferred term (PT) in the latest edition of the MedDRA Glossary of Adverse Drug Reactions (MedDRA 25.1) were used in the present study. MedDRA 25.1 standardizes and minimizes international terminology of terms to describe AEs (14, 15). According to PUBMED age grouping standard (16), the entire population was categorized into three distinct age groups: minors (age < 18 years), adults (18 ≤ age < 65 years), and senior citizens (≥65 years).

### 2.3 Data analyses

Data (e.g., target number of AEs reports and background number of AEs occurrences of the primary suspected drug) were obtained. Potential AE signals were screened based on a four-cell table (14, 17) of the proportional imbalance method (See Supplementary Table S1). Adopt the ROR and MHRA method, the ROR, proportional reporting ratio (PRR), and X^2^ equivalents are calculated, respectively. To avoid false-positive signals, the calculated corresponding values should reach the set threshold and be defined as the PT of valid signals (See Supplementary Table S2) (18–20). The larger the calculated value, the stronger the signal, indicating that the target drug is more likely to be associated with the target AEs, but this does not mean that there is a causal relationship between the two (21). All statistical analyses were undertaken using Prism 8 (GraphPad, La Jolla, CA, United States), SPSS29 and Excel^®^ (Microsoft, Redmond, WA, United States).

## 3 Results

### 3.1 Primary characteristics of AEs reported in the endocrine system

As of the first quarter of 2023, the FAERS database collected 79,700 AEs reports. Among them, there were 22,918, 34,267, 1,239, 13,862, 6,014, and 1,300 AEs reports involving pembrolizumab, nivolumab, cemiplimab, atezolizumab, durvalumab, and avelumab as the primary suspected drug, respectively. Among them, there were 5,322 reports related to the endocrine system (accounting for 6.68% of the total reports), with the aforementioned six drugs accounting for 34.80, 43.71, 1.01, 15.03, 4.17 and 1.28%, respectively. Patients who suffered PD-1/PD-L1 inhibitor-associated AEs were predominantly male (except for those who had AEs after taking pembrolizumab) and most of the population is over aged >65 years. The occupations at the top of the list were physician and consumers. The countries with the largest number of AEs reports by drug were mostly Japan. The specific number and proportion of reports are shown in Table 1.

**Table 1:** Clinical characteristics of reported cases of AEs in endocrine system (*n*, %).

Characteristic	Pembrolizumab	Nivolumab	Cemiplimab	Atezolizumab	Durvalumab	Avelumab
Number of cases	1852	2,326	54	800	222	68
Sex						
Male	860 (46.44)	1,425 (61.26)	15 (27.78)	383 (47.88)	130 (58.56)	41 (60.29)
Female	940 (50.76)	778 (33.45)	3 (5.56)	265 (33.13)	76 (34.23)	26 (38.24)
Unknown	52 (2.81)	123 (5.29)	36 (66.67)	152 (19.00)	16 (7.21)	1 (1.47)
P	0.009	<0.001	0.002	<0.001	<0.001	0.010
Age						
<18 years	2 (0.11)a	5 (0.21)a	0 (0.00)b	1 (0.13)a	0 (0.00)a	0 (0.00)a
18 ≤ years <65	584 (31.53)a	929 (39.94)a	3 (5.56)b	247 (30.88)a	80 (36.04)b	27 (39.71)b
18 ≤ years <65	939 (50.70)a	1,125 (48.37)a	10 (18.52)a	369 (46.13)a	103 (46.40)a	35 (51.47)a
Unknown	327 (17.66)	267 (11.48)	41 (75.93)	183 (22.88)	39 (17.57)	6 (8.82)
*p*	<0.001	<0.001	<0.001	<0.001	<0.001	<0.001
Reporter occupation						
Physician	1,128 (60.91)a	1,492 (64.14)a	44 (81.48)a	665 (83.13)a	158 (71.17)a	63 (92.65)a
Consumer	518 (27.97)a	250 (10.75)a	6 (11.11) a	18 (2.25)a	26 (11.71)a	5 (7.35) b
Other	4 (0.22)a	2 (0.09%)a	0 (0.00)a	0 (0.00)a	0 (0.00)a	0 (0.00)a
Unknown	202 (10.91)	582 (25.02)	4 (7.41)	117 (14.63)	38 (17.12)	0 (0.00)
*p*	<0.001	<0.001	<0.001	<0.001	<0.001	<0.001
Reporting countries (top 5)	Japan (897, 48.43)	Japan (824, 35.43)	USA (19, 35.19)	Japan (381, 47.63)	Japan (104, 46.85)	Japan (25, 36.76)
	USA (452, 24.41)	USA (535, 23.00)	Australia (8,14.81)	USA (94, 12.13)	USA (48, 21.62)	France (9, 13.24)
	France (78, 4.21)	Germany (233, 10.02)	France (7, 12.96)	France (43, 5.38)	France (12, 5.38)	USA (9, 13.24)
	Germany (45, 2.43)	France (228, 9.80)	Korea (5, 9.26)	China (32, 4.00)	Canada (11, 4.95)	Finland (3, 4.41)
	Italy (32, 1.73)	Canada (48, 2.06)	Japan (4, 7.41)	Germany (27, 3.38)	China (10, 4.50)	Britain (3, 4.41)

The categorical variables were tested by *X*^2^, and *p* < 0.05 was statistically significant. When the three groups of data were pairwise compared, *p* < 0.0167, there was a statistical difference, *a* < 0.001, *b* > 0.0167. Unknown groups are not compared.

### 3.2 Proportion of AEs in the endocrine system

**Figure 1:**
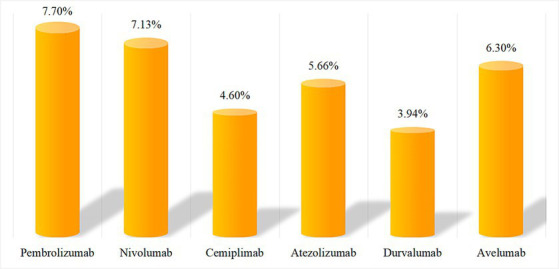
Component ratio of AEs in endocrine system.

After SOC classification of excavated effective signals, we found no significant difference in the ratio of AEs components associated with PD-1/PD-L1 inhibitors in the endocrine system (approximately 3–8%). The specific numbers of cases and composition ratios are shown in Figure 1.

### 3.3 AEs signals and correlation with the endocrine system

**Figure 2:**
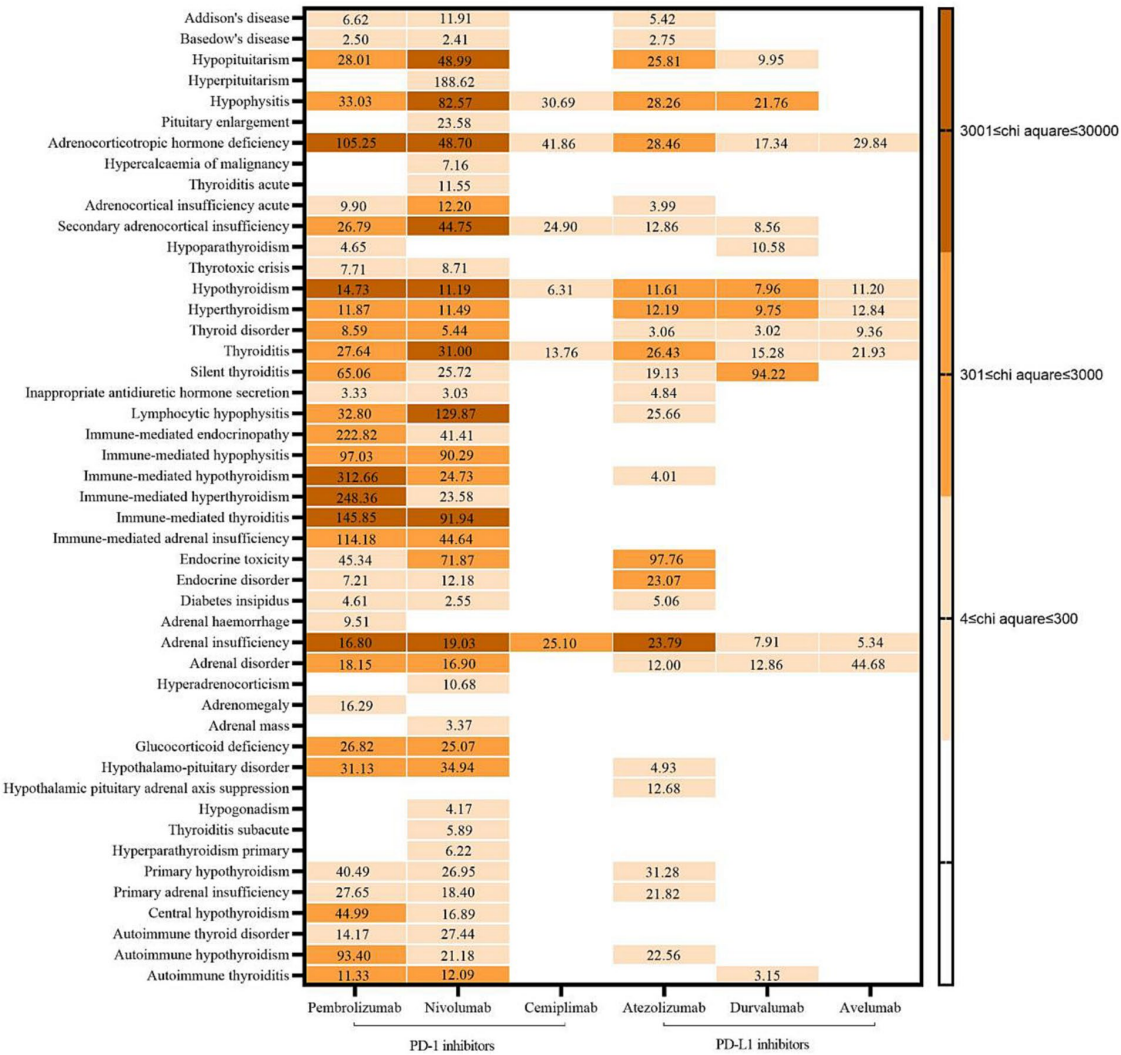
Signals and correlation of adverse events in endocrine system.

A total of 131 AEs signals were detected in the endocrine system: 37 for pembrolizumab, 43 for nivolumab, six for cemiplimab, 25 for atezolizumab, 13 for durvalumab, and seven for avelumab. Endocrine system-related AEs had a strong correlation with pembrolizumab, including immune-mediated hypothyroidism (X^2^ = 27,216.81, ROR = 312.66), adrenocorticotropin deficiency (10,269.46, 105.25), and hypothyroidism (6307.58, 14.73). AEs closely associated with nivolumab were pituitary inflammation (X^2^ = 14,605.84, ROR = 82.57), adrenal insufficiency (6549.80, 19.03), and hypopituitarism (5874.78, 48.99). Adrenal dysfunction was the main factor in AEs attributed to a strong correlation between the use of cemiplimab and atezolizumab (X^2^ = 497.78, ROR = 25.10; X^2^ = 4582.73, ROR = 23.79). The AEs with a strong correlation with durvalumab use was silent thyroiditis (X^2^ = 508.62, ROR = 94.22). The AEs with strong association of avilumab was adrenal disease (X^2^: 252.79, ROR: 44.68). See Figure 2 for details.

### 3.4 Time of occurrence of AEs in the endocrine system

**Figure 3:**
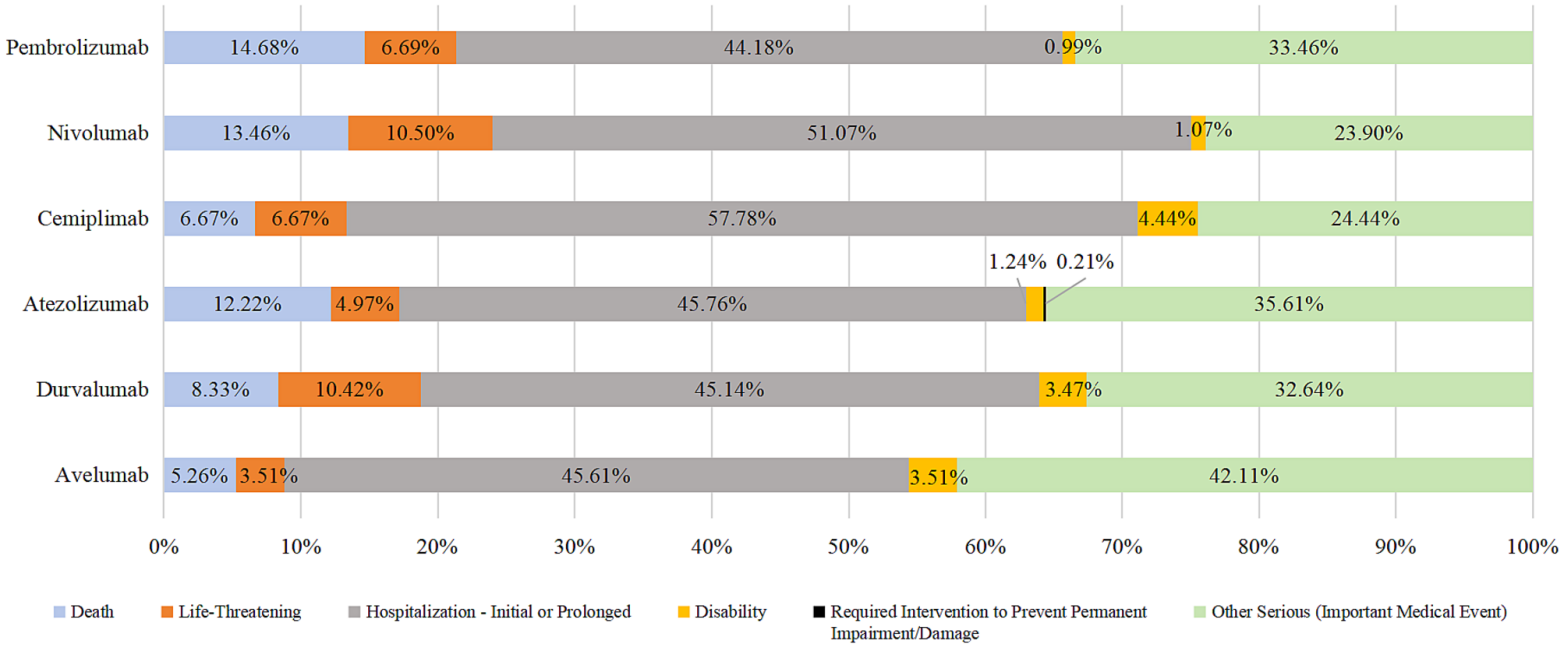
Time distribution of AEs in endocrine system.

The onset time of AEs in the endocrine system was more distributed between 30–365 days, the median time was 61d, the median onset time of AEs in the endocrine system was 42d for pembrolizumab, 63 days for nivolumab, 161 days for cemiplimab, 73.5 days for atezolizumab, and 42 days for durvalumab. Avelumab was 56 days. Time of adverse endocrine system events with the use of six drugs. See Figure 3 for details.

### 3.5 PD-1/PD-L1 inhibitors produce AEs in the endocrine system

**Figure 4:**
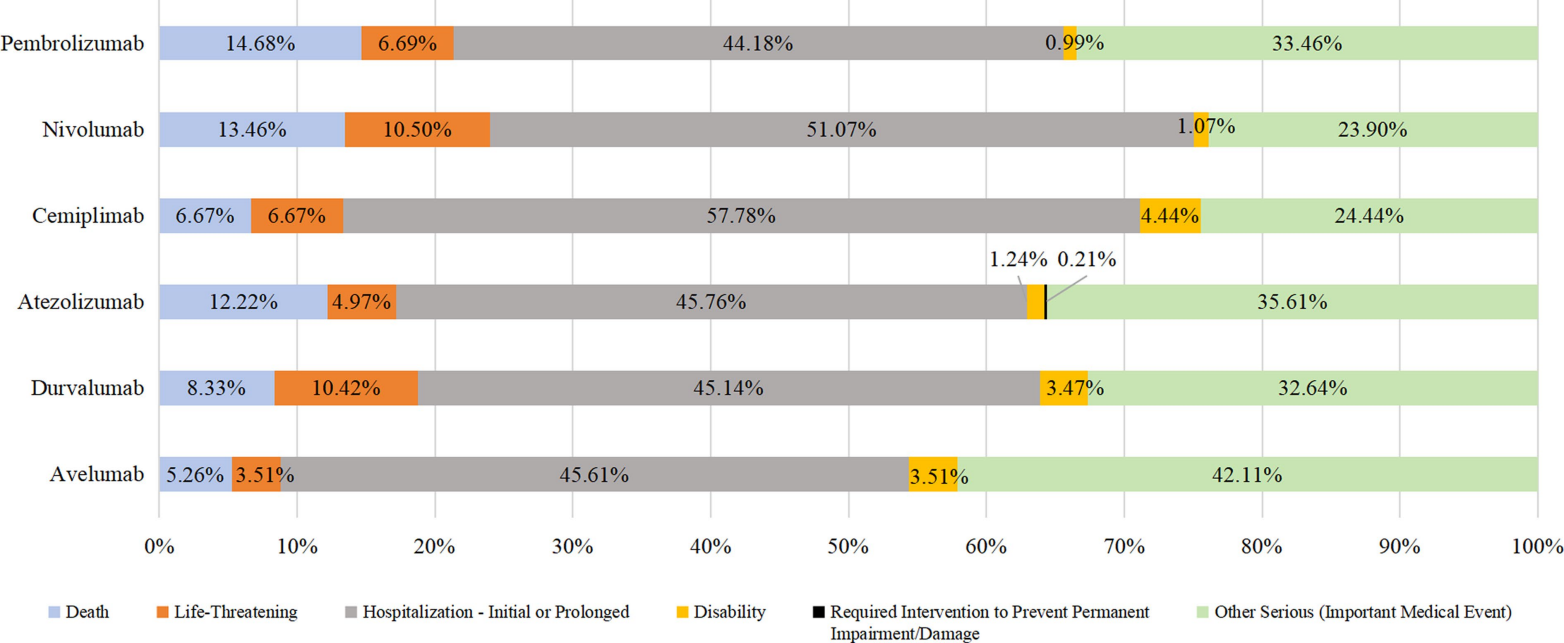
Outcome distribution of AEs in endocrine system.

In addition to unknown serious medical events, the most prevalent outcome of endocrine system AEs due to the use of PD-1/PD-L1 inhibitors was prolonged hospital stay (44.18–57.78%). The other most prevalent outcomes were death, life-threatening injury, and disability. Death due to taking pembrolizumab, nivolumab, or durvalumab accounted for >10% of cases. See Figures 3, 4 for details.

## 4 Discussion

### 4.1 Basic characteristics of reported cases of PD-1/PD-L1 inhibitor-associated endocrine system AEs

We found that, except for pembrolizumab, five PD-1/PD-L1 inhibitors associated with AEs occurred mainly in men, a result which is consistent with those of other studies (22, 23). Our finding is probably related to the completeness of the data. Compared with the data for the other five drugs, the data size for pembrolizumab was relatively large. The proportion of cases in which the sex of the patient was not known was relatively low (2.81%). Results can vary if data on sex are missing. All six PD-1/PD-L1 inhibitors were used for patients aged >65 years, which may have been related to the age when the disease was diagnosed (24, 25), however, elderly men over 65 years of age should be especially aware of the occurrence of relevant AEs. Most of the reporters are medical personnel, indicating that the AEs reported to the database has strong reliability. The countries that reported the most AEs were Japan and the USA. This finding suggests that other countries may be paying less attention to AEs in the endocrine system, but this may also be related to the number of people taking these drugs in such countries.

### 4.2 AEs distribution and correlation in endocrine system associated PD-1/PD-L1 inhibitors

The findings of this study, the number of AEs signals associated with the endocrine system was higher for PD-1 inhibitors than for PD-L1 inhibitors and the degree of correlation was also larger, especially pembrolizumab and nivolumab. The above findings are similar to the results of previous relevant studies (26), and this kind of ADR should be paid attention to when using related drugs. The results also suggest that, which should be considered (especially for pembrolizumab and nivolumab). The main organs involved in AEs associated with pembrolizumab use were the thyroid gland and adrenal glands, whereas they were the pituitary gland and adrenal glands for nivolumab, the adrenal glands for cemiplimab, the adrenal glands for atezolizumab, and the thyroid gland for avelumab. According to the *Clinical Application Guide of Immune Checkpoint Inhibitors for Gynecological Tumors* (2), the common types of endocrine toxicity caused by ICIs are dysfunction in the thyroid gland and acute pituitary inflammation, these data are consistent with the results of our study. As mentioned above, endocrine glands contain rich blood flow and may trigger autoimmune reactions through various ways such as the activation of autoimmune cells, thus involving multiple glands (1). However, the mechanism of adverse reactions in different glands may be different. In addition to immune-mediated mechanisms (3), thyroid injury may also be associated with the upregulation of PD-1 receptors in the thyroid gland (1). Furthermore, elevated levels of serum IL-1β, IL-2, and GM-CSF at baseline, as well as decreased levels of serum IL-8, G-CSF, and MCP-1 at an early stage are correlated with thyroid dysfunction (27, 28). Pituitary is also associated with humoral immunity, usually involving the anterior pituitary, which results in permanent dysfunction of one or more pituitary endocrine axes (29, 30). Combined with the results of our study, the use of PD-1/PD-L1 inhibitors seems to result in additional abnormalities in the functions of the thyroid gland and pituitary gland. These data suggest that monitoring abnormalities in the function of these two organs is important. In the event of a serious acute reaction, immunotherapy should be stopped promptly, and the corresponding drug treatment and symptomatic treatment should be applied (1).

In addition, the above guidelines mentioned that adrenal diseases were rare endocrine toxicity, but the results of this study found that the use of PD-1/PD-L1 inhibitors had a large association with adrenal diseases, which is worth noting. The mechanism of this glandular disease may be related to the infiltration of CD4 + t cells, especially Th1 and Th17 cells (3). Therefore, in clinical practice, medical personnel should still pay attention to the occurrence of such AEs. If patients have abnormal adrenal function and indicators, physicians and clinical pharmacists should judge whether it is caused by such drugs according to the baseline assessment, so as to correctly handle adverse drug reaction (ADR), it also provides reference for whether to adjust the anti-tumor therapy regimen in the future.

### 4.3 Occurrence time and outcome of AEs in endocrine system associated with PD-1/PD-L1 inhibitors

Our data suggested that AEs in the endocrine system occurred between 30 and 365 (mean, 117) days after the use of PD-1/PD-L1 inhibitors, the median time was 61d, which was consistent with Viola suggestion (31). The above results indicate that this type of AEs occurs slowly, and long-term follow-up monitoring is needed for patients using this type of drugs.

Hospitalization/prolonged hospitalization was the most prevalent outcome of AEs, followed by death, life-threatening illness, and disability. According to the Common Terminology Criteria for Adverse Events (CTCAE), patients who reach grade 3 or above will be hospitalized for intravenous hormone therapy (2), especially if there is a corresponding emergency/crisis, Additional treatments, such as anti-infection therapy, blood purification, and ventilatory support, are needed (1), if the treatment is not timely and incorrect, it can endanger life or even death (33), indicating the severity of the AEs, it is necessary to do a baseline assessment before the use of this type of drug in clinical practice, and close monitoring during use to identify ADR as early as possible and timely intervention to reduce or even avoid the occurrence of adverse outcomes. It is worth noting that the death outcome of pembrolizumab, nivolumab and durvalumab accounted for more than 10%. When patients use the above three drugs, clinical pharmacists should set them as key monitoring objects, closely monitor the corresponding indicators and changes in symptoms and signs, pay attention to the suitability of medication, and cooperate with the clinic to improve the prognosis.

## 5 Research limitations

First, the FAERS database has a large amount of data, there are also a lot of missing data information such as gender, age and adverse event occurrence time, especially the time is not accurate enough. Secondly, the accuracy and professionalism of the information of “adverse events” in the database need to be improved. Reporters from different occupational backgrounds may use different descriptions of endocrine toxicity and endocrine disorders, which may lead to deviations in the equivalence calculation of ROR and PRR for a single PT. Despite these limitations, spontaneous reporting may be the best way to collect more AEs that might otherwise be overlooked (34). Third, the AEs signal detected in this study indicates that the drug is statistically correlated with the AEs, but it does not mean that there is a causal link in biology, and further clinical trials are needed to explore (35, 36). In addition, sensitivity analysis cannot be performed in the current proportion imbalance method, and the impact of combined drug use on the outcome is difficult to predict,other research methods can be explored to evaluate the impact in the future. Fourth, due to a lack of information on the total number of people used, the incidence of specific adverse events cannot be calculated (37), therefore, the intensity of the association between drugs and adverse events was measured.

## 6 Conclusion

Men aged ≥65 years should be concerned about endocrine-related AEs. The use of different PD-1/PD-L1 inhibitors mainly involves interaction with the endocrine glands, so physicians should be careful when prescribing drugs for patients with associated underlying diseases. There was a lengthy interval between the use of PD-1/PD-L1 inhibitors and endocrine system-related AEs, but the outcome was serious. Therefore, long-term, meticulous monitoring and appropriate treatment are necessary. Special attention should be given to endocrine system-related AEs when using pembrolizumab, nivolumab, or durvalumab.

## Data availability statement

The datasets presented in this study can be found in online repositories. The names of the repository/repositories and accession number(s) can be found in the article/Supplementary material.

## Ethics statement

Ethical approval was not required for the study involving humans in accordance with the local legislation and institutional requirements. Written informed consent to participate in this study was not required from the participants or the participants' legal guardians/next of kin in accordance with the national legislation and the institutional requirements.

## Author contributions

HS: Conceptualization, Investigation, Validation, Writing – original draft. YH: Visualization, Writing – original draft. SD: Data curation, Writing – original draft. LY: Data curation, Writing – original draft. JW: Data curation, Formal analysis, Writing – review & editing. LC: Methodology, Supervision, Writing – review & editing. ZC: Writing – review & editing.

## Footnotes

^1^ https://dev.mysql.com/doc/refman/8.2/en/
