# Supplementary material for: Endocrine system-related adverse events associated with PD-1/PD-L1 inhibitors: data mining from the FDA adverse event reporting system
Source: Front Med (Lausanne). 2024 Apr 16;11:1366691. doi: 10.3389/fmed.2024.1366691 (PMC11073539; doi:10.3389/fmed.2024.1366691)
Supplement: Supplementary file 1 [file Data_Sheet_1.docx]

Supplementary Material

## Supplementary Tables

**Supplementary Table 1.** Fourfold table of disproportional method.

|  | Adverse event of interest | All other adverse events | Total |
| --- | --- | --- | --- |
| Drug of interest | a | b | a+b |
| All other drugs in FAERS | c | d | c+d |
| Total | a+c | b+d | N= a+b+c+d |

**Supplementary Table 2.** Formulas and thresholds of the ROR method and the MHRA method.

| Method | Formula | Threshold value |
| --- | --- | --- |
| ROR | ROR=ad/bc  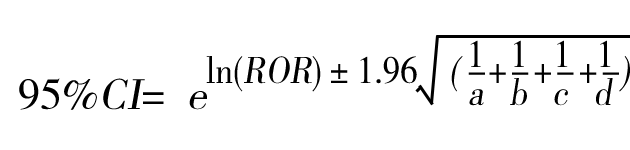SE（lnROR）=$\sqrt{\left（ \frac{1}{a}+\frac{1}{b}+\frac{1}{c}+\frac{1}{d} \right）}$ | a signal is detected, if a≥3,and the lower limit of the 95%(ROR)＞1 |
| MHRA | PRR=$\frac{a/(a+b)}{c/(c+d)}$  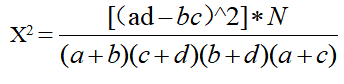 | a signal is detected, if a≥3,and PRR≥2,and X^2^≥4 |

**Supplementary Table 3.** Component ratio of adverse events in endocrine system.

| Effective signal | Pembrolizumab | Nivolumab | Cemiplimab | Atezolizumab | Durvalumab | Avelumab |
| --- | --- | --- | --- | --- | --- | --- |
| Total number of ADEs（n） | 30195 | 41988 | 1087 | 16711 | 6198 | 1032 |
| Number of ADEs in endocrine system（n） | 2326 | 2995 | 50 | 946 | 244 | 65 |
| Component ratio（%） | 7.70 | 7.13 | 4.60 | 5.66 | 3.94 | 6.30 |

**Supplementary Table 4.** PTs of ADEs in endocrine system of six drugs.

| **Pembrolizumab** | | | | | |
| --- | --- | --- | --- | --- | --- |
| PT | a | ROR | lower limit of the 95%(ROR) | PRR | chi-square |
| Addison's disease | 10 | 6.62 | 3.54 | 6.62 | 46.62 |
| Adrenal disorder | 41 | 18.15 | 13.23 | 18.12 | 623.45 |
| Adrenal haemorrhage | 5 | 9.51 | 3.90 | 9.51 | 36.83 |
| Adrenal insufficiency | 261 | 16.80 | 14.82 | 16.62 | 3622.06 |
| Adrenocortical insufficiency acute | 20 | 9.90 | 6.34 | 9.89 | 154.43 |
| Adrenocorticotropic hormone deficiency | 137 | 105.25 | 86.48 | 104.62 | 10269.46 |
| Adrenomegaly | 5 | 16.29 | 6.61 | 16.28 | 67.82 |
| Autoimmune hypothyroidism | 27 | 93.40 | 60.45 | 93.29 | 1854.50 |
| Autoimmune thyroid disorder | 3 | 14.17 | 4.44 | 14.17 | 34.96 |
| Autoimmune thyroiditis | 53 | 11.33 | 8.61 | 11.31 | 478.96 |
| Basedow's disease | 9 | 2.50 | 1.30 | 2.50 | 8.01 |
| Diabetes insipidus | 12 | 4.61 | 2.60 | 4.61 | 33.35 |
| Endocrine disorder | 9 | 7.21 | 3.72 | 7.20 | 46.89 |
| Endocrine toxicity | 4 | 45.34 | 15.78 | 45.33 | 149.50 |
| Glucocorticoid deficiency | 14 | 26.82 | 15.50 | 26.80 | 317.69 |
| Hyperthyroidism | 189 | 11.87 | 10.26 | 11.78 | 1791.18 |
| Hypoparathyroidism | 5 | 4.65 | 1.92 | 4.64 | 14.07 |
| Hypophysitis | 90 | 33.03 | 26.54 | 32.90 | 2494.57 |
| Hypopituitarism | 69 | 28.01 | 21.86 | 27.93 | 1630.91 |
| Hypothalamo-pituitary disorder | 57 | 31.13 | 23.68 | 31.06 | 1494.39 |
| Hypothyroidism | 530 | 14.73 | 13.49 | 14.42 | 6307.58 |
| Immune-mediated adrenal insufficiency | 33 | 114.18 | 76.21 | 114.02 | 2636.12 |
| Immune-mediated endocrinopathy | 22 | 222.82 | 127.46 | 222.61 | 2717.98 |
| Immune-mediated hyperthyroidism | 42 | 248.36 | 164.11 | 247.91 | 5508.45 |
| Immune-mediated hypophysitis | 26 | 97.03 | 62.15 | 96.93 | 1839.18 |
| Immune-mediated hypothyroidism | 185 | 312.66 | 253.70 | 310.14 | 27216.81 |
| Immune-mediated thyroiditis | 57 | 145.85 | 105.95 | 145.49 | 5404.30 |
| Inappropriate antidiuretic hormone secretion | 33 | 3.33 | 2.36 | 3.32 | 53.05 |
| Lymphocytic hypophysitis | 14 | 32.80 | 18.86 | 32.78 | 386.63 |
| Primary adrenal insufficiency | 8 | 27.65 | 13.38 | 27.64 | 187.16 |
| Primary hypothyroidism | 8 | 40.49 | 19.30 | 40.47 | 269.50 |
| Secondary adrenocortical insufficiency | 55 | 26.79 | 20.31 | 26.73 | 1244.82 |
| Secondary hypothyroidism | 10 | 44.99 | 23.08 | 44.97 | 371.05 |
| Silent thyroiditis | 14 | 65.06 | 36.39 | 65.02 | 717.82 |
| Thyroid disorder | 158 | 8.59 | 7.33 | 8.53 | 1021.11 |
| Thyroiditis | 102 | 27.64 | 22.55 | 27.52 | 2376.60 |
| Thyrotoxic crisis | 9 | 7.71 | 3.97 | 7.70 | 51.11 |
| **Nivolumab** | | | | | |
| PT | a | ROR | lower limit of the 95%(ROR) | PRR | chi-square |
| Addison's disease | 26 | 11.91 | 8.01 | 11.90 | 244.24 |
| Adrenal disorder | 56 | 16.90 | 12.85 | 16.87 | 767.56 |
| Adrenal insufficiency | 427 | 19.03 | 17.22 | 18.80 | 6549.80 |
| Adrenal mass | 4 | 3.37 | 1.25 | 3.37 | 6.54 |
| Adrenocortical insufficiency acute | 36 | 12.20 | 8.71 | 12.19 | 347.37 |
| Adrenocorticotropic hormone deficiency | 104 | 48.70 | 39.24 | 48.55 | 3852.18 |
| Autoimmune hypothyroidism | 11 | 21.18 | 11.35 | 21.17 | 190.06 |
| Autoimmune thyroid disorder | 8 | 27.44 | 13.07 | 27.43 | 177.88 |
| Autoimmune thyroiditis | 83 | 12.09 | 9.68 | 12.06 | 791.44 |
| Basedow's disease | 13 | 2.41 | 1.40 | 2.41 | 10.60 |
| Diabetes insipidus | 10 | 2.55 | 1.37 | 2.55 | 9.29 |
| Endocrine disorder | 22 | 12.18 | 7.91 | 12.17 | 211.85 |
| Endocrine toxicity | 8 | 71.87 | 31.83 | 71.85 | 404.72 |
| Glucocorticoid deficiency | 19 | 25.07 | 15.53 | 25.06 | 387.42 |
| Hyperadrenocorticism | 6 | 10.68 | 4.69 | 10.68 | 49.80 |
| Hypercalcaemia of malignancy | 3 | 7.16 | 2.26 | 7.16 | 15.32 |
| Hyperparathyroidism primary | 3 | 6.22 | 1.97 | 6.22 | 12.72 |
| Hyperpituitarism | 3 | 188.62 | 38.07 | 188.60 | 279.91 |
| Hyperthyroidism | 270 | 11.49 | 10.15 | 11.40 | 2418.59 |
| Hypogonadism | 5 | 4.17 | 1.72 | 4.17 | 11.80 |
| Hypophysitis | 262 | 82.57 | 71.39 | 81.95 | 14605.84 |
| Hypopituitarism | 158 | 48.99 | 41.12 | 48.77 | 5874.78 |
| Hypothalamo-pituitary disorder | 90 | 34.94 | 27.90 | 34.85 | 2498.10 |
| Hypothyroidism | 604 | 11.19 | 10.31 | 11.01 | 5205.08 |
| Immune-mediated adrenal insufficiency | 22 | 44.64 | 28.05 | 44.62 | 758.60 |
| Immune-mediated endocrinopathy | 9 | 41.41 | 20.13 | 41.40 | 290.96 |
| Immune-mediated hyperthyroidism | 10 | 23.58 | 12.22 | 23.58 | 192.16 |
| Immune-mediated hypophysitis | 33 | 90.29 | 59.62 | 90.20 | 1969.24 |
| Immune-mediated hypothyroidism | 41 | 24.73 | 17.86 | 24.71 | 824.60 |
| Immune-mediated thyroiditis | 55 | 91.94 | 66.60 | 91.80 | 3322.47 |
| Inappropriate antidiuretic hormone secretion | 45 | 3.03 | 2.26 | 3.03 | 60.30 |
| Lymphocytic hypophysitis | 55 | 129.87 | 92.12 | 129.66 | 4161.24 |
| Pituitary enlargement | 7 | 23.58 | 10.75 | 23.58 | 134.51 |
| Primary adrenal insufficiency | 8 | 18.40 | 8.90 | 18.40 | 119.94 |
| Primary hypothyroidism | 8 | 26.95 | 12.85 | 26.94 | 174.86 |
| Secondary adrenocortical insufficiency | 122 | 44.75 | 36.72 | 44.59 | 4205.22 |
| Secondary hypothyroidism | 6 | 16.89 | 7.33 | 16.89 | 82.32 |
| Silent thyroiditis | 9 | 25.72 | 12.82 | 25.72 | 188.16 |
| Thyroid disorder | 151 | 5.44 | 4.63 | 5.42 | 529.86 |
| Thyroiditis | 162 | 31.00 | 26.25 | 30.86 | 4023.38 |
| Thyroiditis acute | 3 | 11.55 | 3.60 | 11.55 | 27.23 |
| Thyroiditis subacute | 3 | 5.89 | 1.87 | 5.89 | 11.82 |
| Thyrotoxic crisis | 15 | 8.71 | 5.19 | 8.70 | 97.79 |
| **Cemiplimab** | | | | | |
| PT | a | ROR | lower limit of the 95%(ROR) | PRR | chi-square |
| Adrenal insufficiency | 22 | 25.10 | 16.45 | 24.68 | 497.78 |
| Adrenocorticotropic hormone deficiency | 4 | 41.86 | 15.63 | 41.73 | 157.78 |
| Hypophysitis | 5 | 30.69 | 12.72 | 30.57 | 142.21 |
| Hypothyroidism | 13 | 6.31 | 3.65 | 6.25 | 57.39 |
| Secondary adrenocortical insufficiency | 3 | 24.90 | 8.00 | 24.84 | 68.33 |
| Thyroiditis | 3 | 13.76 | 4.43 | 13.73 | 35.32 |
| **Atezolizumab** | | | | | |
| PT | a | ROR | lower limit of the 95%(ROR) | PRR | chi-square |
| Addison's disease | 5 | 5.42 | 2.24 | 5.42 | 17.80 |
| Adrenal disorder | 17 | 12.00 | 7.42 | 11.99 | 167.02 |
| Adrenal insufficiency | 224 | 23.79 | 20.78 | 23.42 | 4582.73 |
| Adrenocortical insufficiency acute | 5 | 3.99 | 1.65 | 3.99 | 11.10 |
| Adrenocorticotropic hormone deficiency | 29 | 28.46 | 19.55 | 28.40 | 722.88 |
| Autoimmune hypothyroidism | 5 | 22.56 | 9.19 | 22.55 | 98.26 |
| Basedow's disease | 6 | 2.75 | 1.23 | 2.75 | 6.64 |
| Diabetes insipidus | 8 | 5.06 | 2.52 | 5.06 | 25.77 |
| Endocrine disorder | 17 | 23.07 | 14.18 | 23.05 | 341.76 |
| Endocrine toxicity | 5 | 97.76 | 37.29 | 97.72 | 396.14 |
| Hyperthyroidism | 119 | 12.19 | 10.15 | 12.09 | 1181.27 |
| Hypophysitis | 49 | 28.26 | 21.17 | 28.17 | 1211.35 |
| Hypopituitarism | 40 | 25.81 | 18.77 | 25.74 | 901.69 |
| Hypothalamic pituitary adrenal axis suppression | 3 | 12.68 | 4.03 | 12.68 | 31.42 |
| Hypothalamo-pituitary disorder | 6 | 4.93 | 2.21 | 4.93 | 18.60 |
| Hypothyroidism | 260 | 11.61 | 10.26 | 11.41 | 2415.50 |
| Immune-mediated hypothyroidism | 3 | 4.01 | 1.29 | 4.01 | 6.72 |
| Inappropriate antidiuretic hormone secretion | 29 | 4.84 | 3.35 | 4.83 | 87.23 |
| Lymphocytic hypophysitis | 7 | 25.66 | 11.99 | 25.65 | 157.24 |
| Primary adrenal insufficiency | 4 | 21.82 | 8.01 | 21.82 | 75.92 |
| Primary hypothyroidism | 4 | 31.28 | 11.37 | 31.27 | 109.89 |
| Secondary adrenocortical insufficiency | 17 | 12.86 | 7.94 | 12.84 | 180.69 |
| Silent thyroiditis | 4 | 26.43 | 9.65 | 26.43 | 92.64 |
| Thyroid disorder | 35 | 3.06 | 2.20 | 3.06 | 48.21 |
| Thyroiditis | 45 | 19.13 | 14.19 | 19.07 | 740.45 |
| **Durvalumab** | | | | | |
| PT | a | ROR | lower limit of the 95%(ROR) | PRR | chi-square |
| Adrenal disorder | 8 | 12.86 | 6.40 | 12.85 | 86.39 |
| Adrenal insufficiency | 34 | 7.91 | 5.64 | 7.87 | 202.61 |
| Adrenocorticotropic hormone deficiency | 8 | 17.34 | 8.62 | 17.32 | 121.09 |
| Autoimmune thyroiditis | 4 | 3.15 | 1.18 | 3.14 | 5.83 |
| Hyperthyroidism | 42 | 9.75 | 7.19 | 9.69 | 324.55 |
| Hypoparathyroidism | 3 | 10.58 | 3.39 | 10.58 | 25.77 |
| Hypophysitis | 17 | 21.76 | 13.45 | 21.70 | 329.14 |
| Hypopituitarism | 7 | 9.95 | 4.73 | 9.94 | 55.81 |
| Hypothyroidism | 79 | 7.96 | 6.37 | 7.87 | 471.09 |
| Secondary adrenocortical insufficiency | 5 | 8.56 | 3.55 | 8.55 | 33.08 |
| Silent thyroiditis | 6 | 94.22 | 40.90 | 94.13 | 508.62 |
| Thyroid disorder | 15 | 3.02 | 1.82 | 3.01 | 20.14 |
| Thyroiditis | 16 | 15.28 | 9.33 | 15.25 | 210.07 |
| **Avelumab** | | | | | |
| PT | a | ROR | lower limit of the 95%(ROR) | PRR | chi-square |
| Adrenal disorder | 6 | 44.68 | 19.97 | 44.48 | 252.79 |
| Adrenal insufficiency | 5 | 5.34 | 2.22 | 5.33 | 17.56 |
| Adrenocorticotropic hormone deficiency | 3 | 29.84 | 9.58 | 29.77 | 82.93 |
| Hyperthyroidism | 12 | 12.84 | 7.27 | 12.73 | 129.50 |
| Hypothyroidism | 24 | 11.20 | 7.48 | 11.01 | 218.35 |
| Thyroid disorder | 10 | 9.36 | 5.02 | 9.29 | 73.92 |
| Thyroiditis | 5 | 21.93 | 9.09 | 21.85 | 99.04 |

**Supplementary Table 5.** Time distribution of ADEs in endocrine system (n,%)

| Time | Pembrolizumab | Nivolumab | Cemiplimab | Atezolizumab | Durvalumab | Avelumab |
| --- | --- | --- | --- | --- | --- | --- |
| T≤1 d | 41(9.56%) | 43(5.55%) | 0(0.00%) | 14(4.07%) | 1(1.59%) | 1(2.86%) |
| 1＜T≤7 d | 44(10.26%) | 39(5.03%) | 0(0.00%) | 24(6.98%) | 3(4.76%) | 2(5.71%) |
| 7＜T≤30 d | 105(24.48%) | 163(21.03%) | 2(6.06%) | 71(20.64%) | 20(31.75%) | 9(25.71%) |
| 30＜T≤365 d | 211(49.18%) | 479(61.81%) | 25(75.76%) | 215(62.50%) | 35(55.56%) | 20(57.14%) |
| T＞365 d | 28(6.53%) | 51(6.58%) | 6(18.18%) | 20(5.81%) | 4(6.35%) | 3(8.57%) |
| Median time（d） | 42 | 63 | 161 | 73.5 | 42 | 56 |

**Supplementary Table 6.** Outcome distribution of ADEs in endocrine system

| Outcome | Pembrolizumab | Nivolumab | Cemiplimab | Atezolizumab | Durvalumab | Avelumab |
| --- | --- | --- | --- | --- | --- | --- |
| Death | 193(14.68%) | 227(13.46%) | 3(6.67%) | 59(12.22%) | 12(8.33%) | 3(5.26%) |
| Life-Threatening | 88(6.69%) | 177(10.50%) | 3(6.67%) | 24(4.97%) | 15(10.42%) | 2(3.51%) |
| Hospitalization - Initial or Prolonged | 581(44.18%) | 861(51.07%) | 26(57.78%) | 221(45.76%) | 65(45.14%) | 26(45.61%) |
| Disability | 13(0.99%) | 18(1.07%) | 2(4.44%) | 6(1.24%) | 5(3.47%) | 2(3.51%) |
| Required Intervention to Prevent Permanent Impairment/Damage | 0(0.00%) | 0(0.00%) | 0(0.00%) | 1(0.21%) | 0(0.00%) | 0(0.00%) |
| Other Serious (Important Medical Event) | 440(33.46%) | 403(23.90%) | 11(24.44%) | 17(35.61%) | 47(32.64%) | 24(42.11%) |
